# Proteomics-based biomarkers of plasma exosomes from patients with acute myocardial infarction

**DOI:** 10.1371/journal.pone.0343804

**Published:** 2026-03-10

**Authors:** Yuye Wang, Haoran Li, Wanning Mou, Jiang Zhu

**Affiliations:** 1 Department of Anesthesiology, The Second Affiliated Hospital of Soochow University, Suzhou, China; 2 Department of Emergency Medicine, The Second Affiliated Hospital of Soochow University, Suzhou, China; Second Xiangya Hospital, CHINA

## Abstract

**Purpose:**

This study aimed to identify plasma exosomal protein biomarkers for early recognition of acute myocardial infarction (AMI) using label-free quantitative proteomics.

**Methods:**

Patients presenting to the Second Affiliated Hospital of Soochow University with chest discomfort between February 2022 and December 2022 were enrolled. Plasma exosomes from a discovery cohort (six AMI patients and six non-AMI controls) were analyzed by mass spectrometry. Differentially expressed proteins (DEPs) were subjected to Gene Ontology (GO) and Kyoto Encyclopedia of Genes and Genomes (KEGG) pathway analyses. Candidate proteins were further validated by western blot (WB) in an independent validation cohort (20 AMI patients and 20 non-AMI controls). Receiver operating characteristic (ROC) curve analysis was performed to evaluate the diagnostic potential of the DEPs for AMI.

**Results:**

A total of 24 DEPs were identified, of which 11 were upregulated and 13 were downregulated in AMI patients. WB analysis confirmed the significantly elevated expression of Inter-alpha-trypsin inhibitor heavy chain H4 (ITIH4) in AMI-derived exosomes. The area under the ROC curve (AUC) for ITIH4 was 0.8825, indicating high diagnostic accuracy. Correlation analysis revealed a significant positive correlation between plasma exosomal ITIH4 levels and the level of cTnI expression (R = 0.702, *P* < 0.001), indicates that exosomal ITIH4 may serve as a novel acute-phase reactant during myocardial injury.

**Conclusion:**

Plasma exosomal ITIH4 is closely associated with AMI and may serve as a promising early diagnostic biomarker.

**Trial registration:**

Registered at the Chinese Clinical Trial Registry (https://www.chictr.org.cn/showproj.html?proj=149623) with No. ChiCTR2200056343 on February 4, 2022. Principal investigator: Jiang Zhu.

## 1. Introduction

Acute myocardial infarction (AMI) is a severe form of acute coronary syndrome (ACS) caused by acute and sustained occlusion of the coronary arteries. This leads to myocardial ischemia, hypoxia, and necrosis [1]. Reperfusion strategies have significantly improved the prognosis of AMI patients. Nevertheless, AMI remains one of the leading causes of mortality worldwide [2].

Early and efficient coronary reperfusion is the main therapeutic goal in AMI, especially ST-segment elevation myocardial infarction (STEMI). This can be achieved through percutaneous coronary intervention or thrombolytic therapy [3]. Although the cardiac troponin T assay, the gold standard for diagnosing AMI, has improved diagnostic accuracy and reduced mortality, it can yield false-positive results among patients with stable coronary artery disease or apparently healthy individuals [4].

Symptoms suggestive of ACS are a major cause for emergency department visits worldwide [5]. However, only a small fraction of these patients are ultimately diagnosed with ACS. Hsia analyzed 10,907 patients presenting with chest pain as the primary complaint, and found that 51.7% were diagnosed with nonspecific chest pain, whereas only 5.1% had confirmed ACS [6]. Among patients with AMI, a substantial proportion (up to 15%) had myocardial infarction with nonobstructive coronary arteries (MINOCA). These patients present with typical AMI symptoms, elevated cardiac biomarkers, and ischemic electrocardiographic changes, however, coronary angiography reveals no significant stenosis (<50%) [7]. For patients with chest pain who are highly suspected of AMI, such as those with aortic dissection, inappropriate administration of thrombolytic therapy or invasive coronary angiography may lead to fatal outcomes. Thus, the development of highly sensitive and specific biomarkers is crucial for early detection of AMI.

Exosomes are nanoscale extracellular vesicles secreted by living cells that maintain cellular topological characteristics and contain diverse bioactive components including proteins, RNAs, and DNAs [8]. The intact phospholipid bilayer membrane effectively shields the molecular cargo from extracellular degradation. Exosomes detectable in diverse biological fluids demonstrate cell-specific responses to cellular activation and damage [9,10]. These unique properties make exosomes promising clinical biomarkers for disease diagnosis, progression monitoring, and prognostic assessment [11].

Notably, emerging evidence has revealed the pivotal role of exosomes in modulating molecular pathways associated with AMI pathogenesis [8]. Plasma exosomal miR-208a is significantly upregulated in ACS and is crucial for its diagnosis [12]. Researchers have observed markedly elevated levels of exosomal miR-126 and miR-21 in the serum of patients with unstable angina and AMI. Notably, circulating exosomal miR-126 levels are positively correlated with the degree of coronary artery stenosis [13]. Zhao identified significantly elevated exosomal miR-183 levels in AMI patients, indicating its potential as a novel diagnostic biomarker [14]. Several studies have shown that injured cardiomyocytes release exosomes that are enriched with cardiac-specific miRNAs [15]. Exosomal proteomics reveals conserved core proteins along with cell/tissue-specific repertoires [16]. Li et al. identified three proteins (polygenic immunoglobulin, cystatin C, and C5a) independently associated with ACS via extracellular vesicle proteomics [17]. Separately, exosomal Cyr61 discriminates unstable angina (UA)/AMI/ACS patients from healthy individuals [18]. Yao further identified PLG, C8B, and F2 as potential biomarkers of AMI [19]. Cheow reported 252 differentially expressed proteins (DEPs) between AMI and UA exosomes, including upregulated platelet basic protein, glycoprotein Iα chain, apolipoprotein D, and apolipoprotein C-III [20].

Based on these findings, we hypothesized that plasma exosomes exhibit distinct protein expression profiles in AMI and non-AMI controls. These DEPs can be identified using label-free quantitative proteomics and potentially serve as diagnostic biomarkers for AMI.

## 2. Materials and methods

### 2.1. Study subjects and grouping

This study enrolled patients who presented to the Second Affiliated Hospital of Soochow University between February 2022 and December 2022 with complaints of chest discomfort. The research design incorporated both the screening and validation cohorts. The inclusion criteria were as follows: (1) presence of chest pain or other cardiac symptoms, (2) documented ST-segment alterations on electrocardiography, (3) abnormal cardiac biomarkers, including troponin elevation, and (4) clinical indication for coronary angiography. Exclusion criteria were: (1) prior history of severe cardiovascular or cerebrovascular disease, (2) active malignancy, and (3) history of major surgery within the previous six months. Participants were stratified into two groups based on coronary angiography findings [21]. The acute myocardial infarction (AMI) group consisted of patients demonstrating significant coronary artery occlusion (≥70% stenosis) requiring percutaneous coronary intervention. The non-AMI control group (CON), patients with negative coronary angiography, consisted of patients who underwent coronary angiography for chest pain but had normal coronary anatomy or clinically nonsignificant stenosis (<50%). The study protocol was approved by the Institutional Ethics Committee of the Second Affiliated Hospital of Soochow University (Approval No. JD-LK-2021-117-02). All participants provided written informed consent prior to study enrollment.

### 2.2. Plasma sample collection

Arterial blood samples were collected via radial artery catheterization using coronary angiography catheters and immediately transferred to EDTA-coated anticoagulant tubes. After centrifugation at 3,000 × g for 15 min (4°C), the plasma supernatant was isolated and cryopreserved at −80°C for subsequent analysis.

### 2.3. Exosome isolation

Plasma-derived exosomes were isolated by ultracentrifugation. Briefly, the frozen plasma samples were thawed at 37°C in a water bath and subjected to sequential centrifugation. Initial centrifugation was performed at 2,000 × g for 30 min (4°C) to remove cellular debris. This was followed by centrifugation at 12,000 × g for 45 min (4°C) to eliminate large extracellular vesicles. The clarified supernatant was re-suspended in 20 mL of ice-cold phosphate-buffered saline (PBS) and filtered through a 0.45 μm pore-size membrane. The filtrate was subjected to ultracentrifugation at 110,000 × g for 2 h (4°C) using fixed-angle rotors. After discarding the supernatant, the pellet was reconstituted in 3 mL ice-cold PBS and subjected to a repeat ultracentrifugation step under identical conditions. The final exosomal pellet was resuspended in 100 μL ice-cold PBS and aliquoted for downstream analyses: 10 μL for transmission electron microscopy (TEM), 10 μL for nanoparticle tracking analysis (NTA), and 30 μL for western blotting (WB). The remaining aliquots were processed for mass spectrometric analysis, following standard tryptic digestion protocols.

### 2.4. Nanoparticle tracking analysis

For nanoparticle size analysis, 5 μL of isolated plasma exosomes was diluted to 300 μL with PBS and gently mixed by pipetting. The samples were then transferred to a dedicated cuvette. The size distribution was quantified using a Zetasizer Nano S analyzer following the manufacturer’s protocol. Measurements were performed in triplicate, and hydrodynamic diameter data were processed using the NanoSight NTA software.

### 2.5. Transmission electron microscopy

Exosome morphology was observed using transmission electron microscopy with negative staining. Briefly, a 10 μL aliquot of plasma exosomes was diluted to 20 μL using PBS (filtered through a 0.22 μm membrane). The exosome suspension (10 μL) was added dropwise to a carbon-coated copper grid and allowed to settle for 5 min. The excess liquid was carefully removed using filter paper, and the grid was air-dried under a warm lamp. Subsequently, 10 μL of phosphotungstic acid was added for negative staining, followed by drying under a warm lamp. The samples were observed using a Transmission Electron Microscope.

### 2.6. Western blot

Plasma exosomal protein concentrations in the samples were quantified using a BCA assay. Subsequently, 20 μg of protein was loaded and transferred onto a PVDF membrane, which was then blocked with 5% skim milk in TBST for 1 h. Membranes were incubated with primary antibodies overnight at 4°C in TBST containing 5% BSA. After incubation, the membrane was washed thrice with TBST (10 min each wash). The membrane was then incubated with secondary antibodies at room temperature for 1 h in 5% skim milk in TBST under gentle shaking. Finally, protein expression was visualized using chemiluminescence reagents and a gel imaging system.

### 2.7. Mass spectrometry analysis

**Fig 1:**
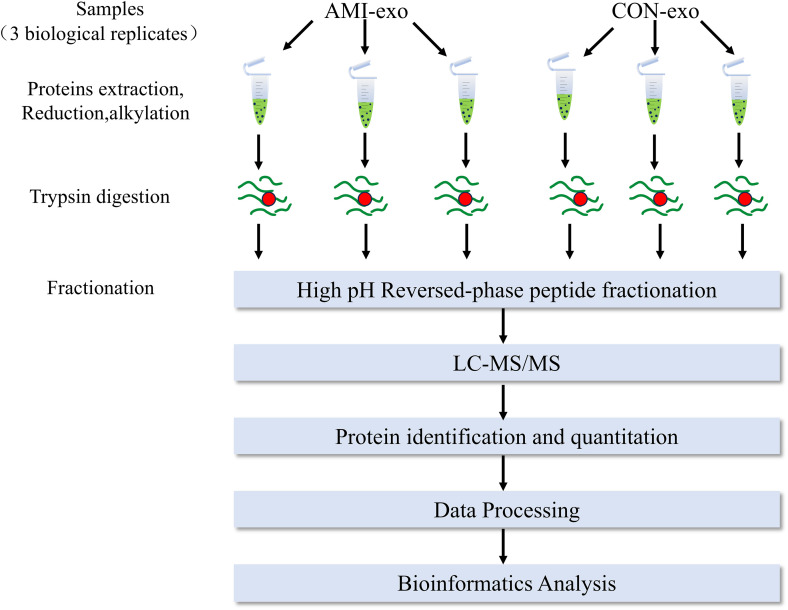
Mass spectrometry flowchart.

Mass spectrometry analysis was performed in accordance with the operating protocols of the liquid chromatography system and the mass spectrometer (S1 Text). The experimental workflow (Fig 1) included (1) sample preparation, (2) protein extraction, (3) protein digestion, (4) chromatographic fractionation, (5) LC-MS/MS data acquisition, and (6) database searching(supplement).

### 2.8. Cluster analysis

Hierarchical clustering was performed using Cluster 3.0 and visualized with Java Treeview. Clustering utilized the Euclidean distance metric and average linkage method. The results were displayed as a heatmap and a dendrogram.

### 2.9. GO annotation

Protein sequence annotation was performed using Blast2GO software. First, the identified proteins were aligned against a database to obtain homologous protein functional information. Subsequently, Gene Ontology (GO) terms associated with the aligned sequences were extracted and qualified GO terms were annotated as differentially expressed proteins (DEPs). For sequences lacking annotation, InterProScan was used for supplementary annotation.

### 2.10. KEGG annotation

Kyoto Encyclopedia of Genes and Genomes (KEGG) pathway analysis was performed using the KEGG database. Following annotation, the protein sequences were blasted against the KEGG database to obtain KEGG Orthology identifiers and were mapped to pathways. KEGG pathway annotation for DEPs was conducted using KEGG Automatic Annotation Server.

### 2.11. Enrichment analysis

Enrichment analysis was performed based on Fisher’s exact test, considering all quantified proteins as the background dataset. The Benjamini-Hochberg correction for multiple testing was further applied to adjust *P*-values. Only functional categories and pathways with *P*-values below a threshold of 0.05 were considered statistically significant.

### 2.12. Statistical analysis

Plasma exosomal protein levels between patients with AMI and non-AMI controls were compared using the Mann-Whitney U test in GraphPad Prism 8. DEPs were identified using thresholds of a false discovery rate < 0.05 (adjusted using the Benjamini-Hochberg method) and a |fold change| ≥ 2. The degree of correlation between cardiac troponin I (cTnI) and DEPs was analyzed using Spearman’s correlation coefficient. The diagnostic potential of candidate DEPs was evaluated using receiver operating characteristic (ROC) curve analysis, reporting the area under the curve (AUC) with 95% confidence intervals, sensitivity, and specificity. Statistical significance was defined as *P* < 0.05 (two-tailed). Fisher’s exact test was used, where appropriate.

## 3. Results

### 3.1. General experiment design

**Fig 2:**
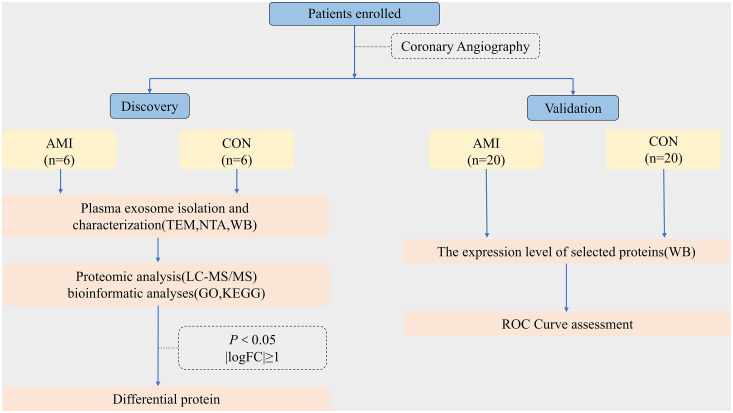
Experimental flowchart. Plasma samples from AMI and non-AMI patients were collected for exosome isolation. LC-MS/MS-based proteomics was performed to characterize exosomal protein profiles and identify differentially expressed proteins between groups.

To obtain comprehensive knowledge of proteins in plasma exosomes from patients with AMI, we designed a strategy based on proteomic analysis (Fig 2). In the discovery phase, plasma samples were collected from patients diagnosed with AMI (n = 6) and non-AMI controls (n = 6). Exosomes were isolated via ultracentrifugation and characterized by TEM, NTA, and WB. Next, proteomic analysis based on LC-MS/MS was performed to identify the protein profiles of the exosome samples and determine the exosomal proteins that were differentially expressed between AMI and non-AMI controls. Bioinformatic analyses, including GO function enrichment analysis and KEGG pathway enrichment analysis, were performed on DEPs. After bioinformatic analysis, candidate proteins were selected for further validation. In the validation phase, exosomes were isolated from individual plasma samples of 20 AMI and 20 non-AMI controls. The expression levels of the candidate proteins were validated by WB. Significant differences existed between the groups regarding smoking history, WBC count, RBG, LDL and cTnI levels. However, no significant differences were observed in the other baseline characteristics or laboratory parameters. The findings were comparable between the two groups (Table 1).

**Table 1:** Clinical characteristics of the patients recruited.

Variables	Discovery		Validation	
	CON (n = 6)	AMI (n = 6)	CON (n = 20)	AMI (n = 20)
**Gender (male/female)**	3/3	6/0	13/7	18/2
**Age (years)**	64.33 ± 8.80	54.67 ± 14.76	56.35 ± 13.80	60.30 ± 13.85
**Smoking, n (%)**	2 (33.33)	5 (83.33)	7 (35)	15 (75)^*^
**Alcohol use, n (%)**	2 (33.33)	5 (83.33)	6 (30)	5 (25)
**Hypertension, n (%)**	3 (50)	5 (83.33)	7 (35)	11 (55)
**WBC (10** ^**9**^ **/L)**	5.97 ± 2.21	10.20 ± 2.97^*^	6.70 ± 2.15	9.97 ± 3.09^**^
**CRP (mg/L)**	4.68 ± 0.19	4.1 ± 3.08	7.21 ± 8.71	32.37 ± 69.52
**RBG (mmol/L)**	4.76 ± 0.35	5.45 ± 0.36^**^	5.24 ± 0.86	6.36 ± 1.31^**^
**TG (mmol/L)**	1.14 ± 0.39	2.07 ± 1.27	1.60 ± 0.96	1.77 ± 0.74
**TC (mmol/L)**	3.47 ± 0.53	4.00 ± 0.57	3.96 ± 0.91	4.58 ± 1.03
**HDL (mmol/L)**	1.04 ± 0.32	0.93 ± 0.21	1.10 ± 0.29	0.93 ± 0.29
**LDL (mmol/L)**	2.06 ± 0.38	2.59 ± 0.37^*^	2.32 ± 0.73	3.03 ± 0.82^**^
**cTnI (ng/mL)**	0.03 ± 0.01	20.13 ± 20.30^*^	0.06 ± 0.14	25.53 ± 20.29^**^

Compared with the CON group, ^*^P < 0.05, ^**^P < 0.01, WBC White Blood Cell, CRP C-Reactive Protein, RBG Random Blood Glucose, TG Triglycerides, TC Total Cholesterol, HDL High-Density Lipoprotein, LDL Low-Density Lipoprotein, cTnI cardiac troponin I.

### 3.2. Isolation and identification of exosomes

**Fig 3:**
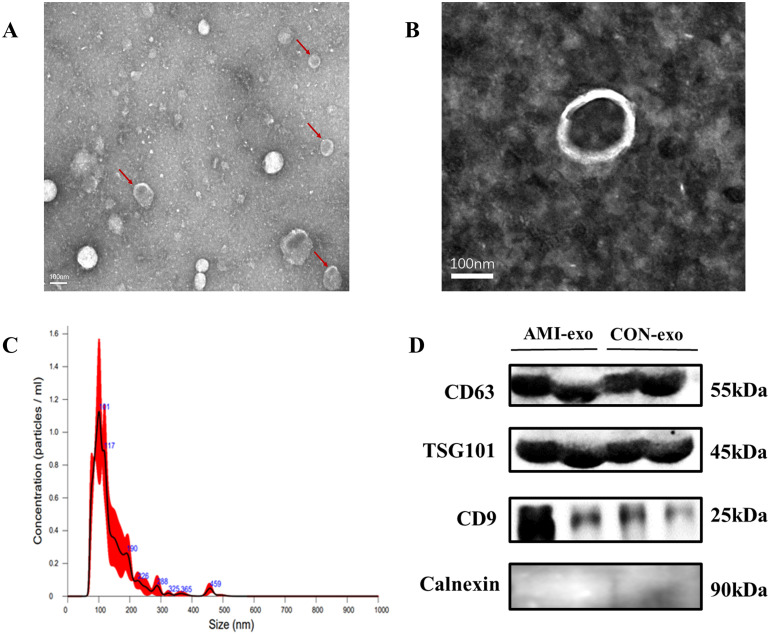
Characterization of plasma-derived exosomes. **(A, B)** Transmission electron microscope images of purified exosomes isolated from non-AMI and AMI patients. Scale bars: 100 nm. **(C)** Size distribution of plasma exosomes by nanoparticle tracking analysis. **(D)** Expression of exosomal markers (CD9, CD63, TSG101) was analyzed by western blot. AMI, acute myocardial infarction; TSG101, tumor susceptibility 101.

Exosomes from the plasma samples were isolated by ultracentrifugation. TEM, NTA, and WB analyses were performed on exosomes isolated from plasma samples. Plasma exosomes were observed under transmission electron microscopy to be in the form of sunken cakes, with a cup-shaped morphology, intact membranous structure, and diameters ranging from 30 to 150 nm (Figs 3A and 3B). NTA showed that the mean size of purified exosomes was 138.7 nm, and the primary peak size was 100.7 nm, which was consistent with the exosome diameter characteristics (Fig 3C, S1 Fig). WB analysis revealed that exosomal marker proteins (TSG101, CD63, and CD9) were significantly expressed in exosome samples. Conversely, calnexin (a marker of intracellular protein contamination) was absent (Fig 3D). Altogether, these results indicate that plasma exosomes were successfully isolated from clinical plasma samples in this study.

### 3.3. Proteome profile and differential proteins

**Fig 4:**
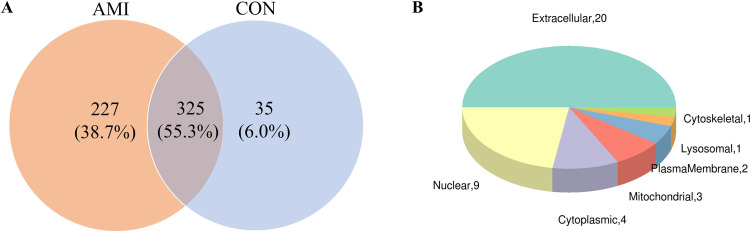
Proteomic analysis of plasma-derived exosomes. **(A)** Venn diagram of proteins detected in AMI and non-AMI groups. **(B)** Subcellular localization of DEPs.

**Fig 5:**
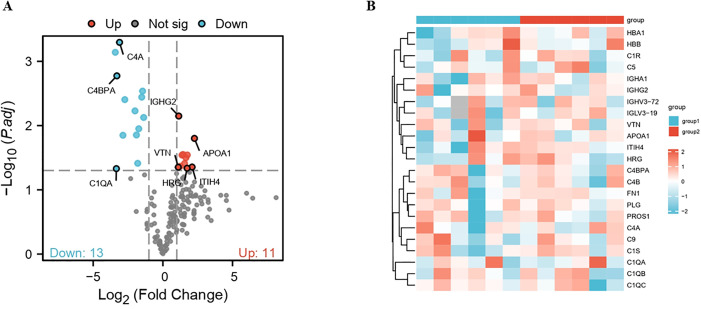
Identification of DEPs analysis. **(A)** Volcano plot of DEPs. Downregulated proteins (light blue); upregulated proteins (red); non-significant proteins (gray). **(B)** Hierarchical clustering of DEPs. Red: upregulated proteins; blue: downregulated proteins; white: missing quantitative data.

In the present study, proteomic profiling was performed to identify DEPs between AMI and non-AMI controls. The mass spectrometry proteomics data have been deposited to the ProteomeXchange Consortium (https://proteomecentral.proteomexchange.org) via the iProX partner repository with the dataset identifier PXD070870 [22,23]. A total of 587 unique proteins were identified, of which 325 were present in both samples (Fig 4A). Additionally, we predicted the subcellular localization of all identified proteins using Wolfpsort. Most identified proteins (83.3%) were located in the extracellular region (Fig 4B). A fold change ≥ 2 and *P*-value < 0.05 was set as the criterion for the selection of upregulated or downregulated genes. Finally, 24 genes were screened as DEPs, of which 11 and 13 proteins were upregulated and downregulated, respectively, in the AMI group. Notably, the Immunoglobulin alpha-2 heavy chain protein in the AMI group lacked complete annotation information, including the gene name designation (Table 2). The differential protein expression patterns between groups were visualized using volcano plot analysis (Fig 5A). Hierarchical clustering analysis was performed to validate the group separation, demonstrating distinct clustering patterns between AMI and non-AMI controls. The clustering results indicated that the identified DEPs effectively distinguished between the two groups, although individual sample variability was observed, reflecting the inherent biological heterogeneity (Fig 5B).

**Table 2:** Information of identified DEPs.

Protein accession	Gene name	FC^a^	Protein accession	Gene name	FC^a^
P02647	APOA1	4.808	P00747	PLG	0.384
Q14624	ITIH4	4.282	P00736	C1R	0.364
A0A0B4J1Y9	IGHV3–72	3.368	P02751	FN1	0.35
P04196	HRG	3.345	P02748	C9	0.301
P69905	HBA1	3.147	P09871	C1S	0.285
P68871	HBB	2.945	P07225	PROS1	0.269
P01876	IGHA1	2.727	P01031	C5	0.25
P01714	IGLV3–19	2.597	P02746	C1QB	0.15
P0DOX2		2.388	P02747	C1QC	0.137
P01859	IGHG2	2.203	P0C0L4	C4A	0.116
P04004	VTN	2.157	P04003	C4BPA	0.101
			P02745	C1QA	0.099
			P0C0L5	C4B	0.094

FC: fold change, ^a^ AMI vs non-AMI control.

### 3.4. GO and KEGG functional annotation

**Fig 6:**
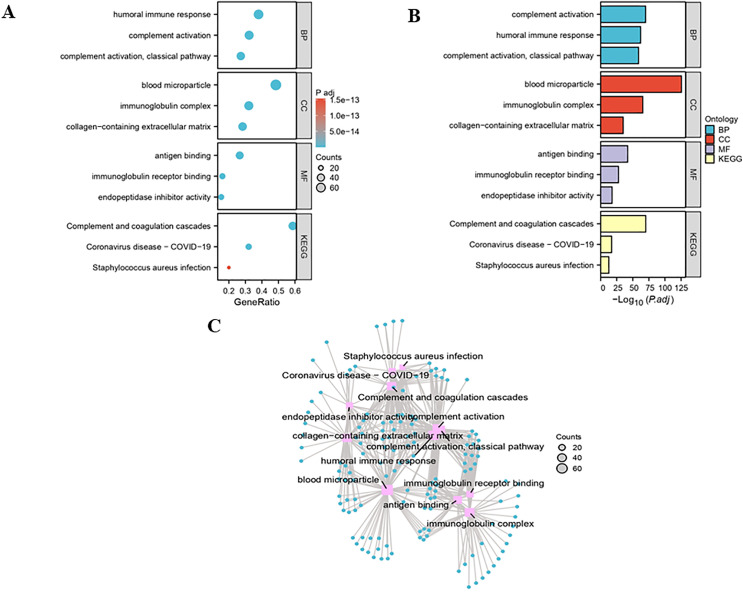
GO and KEGG analysis of DEPs. **(A)** Bubble plot of significantly enriched GO terms and KEGG pathways. The gene ratio represents the proportion of DEPs mapped to a specific term. Bubble size corresponds to the number of enriched genes (Count), and color intensity indicates the statistical significance (-log10 (adjusted P-value)). **(B)** Bar plot displaying the top significantly enriched terms ranked by their statistical significance (-log10 (adjusted P-value)). **(C)** Network visualization showed the interrelationships among the top enriched GO terms and KEGG pathways. Nodes represent functional terms, and edges connect closely related terms, highlighting core biological themes.

To gain insights into the potential biological functions of the differentially expressed genes, we performed comprehensive GO and KEGG pathway enrichment analyses (Figs 6A and 6B). The results consistently revealed significant enrichment of terms related to immune and inflammatory responses. Specifically, the most prominent GO biological process terms included “humoral immune response”, “complement activation”, “complement activation, and classical pathway”. At the cellular component level, genes were predominantly enriched in “blood microparticle” and “immunoglobulin complex”, while molecular functions were primarily associated with “antigen binding” and “immunoglobulin receptor binding”. KEGG pathway analysis further substantiated these findings, identifying “Complement and coagulation cascades”, “Coronavirus disease – COVID-19”, and “Staphylococcus aureus infection” as the most significantly enriched pathways. Network visualization confirmed the close interrelationships among these immune-related terms and pathways, highlighting a coherent functional theme. Collectively, these enrichment analyses suggested that the identified genes were critically involved in host immune and inflammatory processes (Fig 6C).

### 3.5. Experimental validation of candidate biomarkers

**Fig 7:**
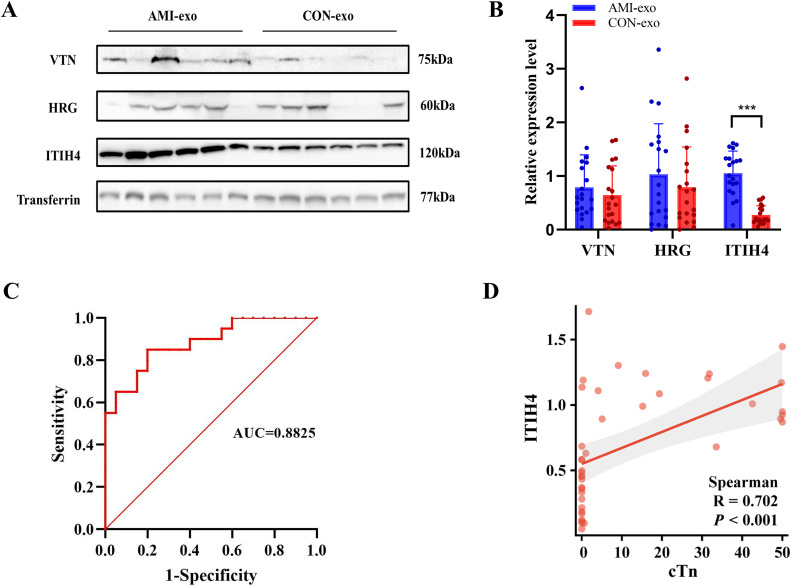
Experimental validation of candidate DEPs. **(A)** Western blot analysis of ITIH4, VTN, and HRG. **(B)** Quantification of protein expression. **(C)** ROC curve analysis of ITIH4. **(D)** Scatter plot of the correlation between plasma exosomal ITIH4 and cTnI. ^***^P < 0.001.

ITIH4, VTN, and HRG were choosed as candidate biomarkers for AMI validation, primarily based on their significant upregulation in our proteomic analysis, enrichment in AMI-relevant pathways, and supporting evidence from prior studies related to systemic inflammation and cardiovascular events. The same inclusion and exclusion criteria were used to collect 20 plasma samples from patients with AMI, and 20 plasma samples from patients in the non-AMI control group. Transferrin served as an internal reference for the plasma secretory proteins. As shown in Fig 7A, HRG and VTN expression levels showed no significant differences between the groups, whereas ITIH4 was markedly upregulated in AMI (*P* < 0.001, Fig 7B), consistent with the proteomic findings. To evaluate the diagnostic potential of ITIH4 for AMI, receiver operating characteristic (ROC) curve analysis of plasma exosomal ITIH4 levels in both patients with AMI and controls was conducted. The analysis revealed an area under the curve (AUC) of 0.8825 (95% confidence interval: 0.7804–0.9846) for ITIH4 (Fig 7C). Furthermore, the correlation between plasma exosomal ITIH4 and plasma cTnI were evaluated. As shown in Fig 7D, ITIH4 expression level was significantly positive correlated with the cTnI expression level. These results demonstrated that plasma exosomal ITIH4 exhibits substantial discriminative capacity for AMI identification, suggesting its potential clinical utility as a diagnostic biomarker.

## 4. Discussion

Quantitative methods, including ultrasensitive mass spectrometry, have been one of the most widely used biomarker discovery methods in recent years [24]. Label-free quantitative proteomics has been widely used for the discovery of potential biomarkers in cardiovascular and other diseases [25].

According to a report presented by the Global Burden of Disease Study, in 2017 these diseases were responsible for 31.8% of all deaths worldwide, and at least half of all cardiovascular deaths were caused by ischemic heart diseases [26]. As the main cause of death among ischemic heart diseases worldwide, being part of the ACS and representing a high impact on morbidity and cost to society, there is a need to develop strategies to detect any signs of AMI in time. In recent years, electrocardiographic (ECG) investigations have been the main method for establishing AMI diagnosis, but only 57% of AMI patients show ECG changes; therefore, clinical evaluations combined with the use of an ECG are insufficient to diagnose AMI in most patients with ACS in the emergency department [27]. Therefore, measurement of the levels of biomarkers in plasma (blood assays) is one of the most applied strategies for evaluating the prognosis of AMI, as some of them are specifically related to a heart attack. Currently, troponin I and troponin T are commonly used biomarkers of myocardial injury [28]. In addition to ACS, conditions such as pulmonary embolism, renal failure, heart failure, myocarditis, arrhythmias, trauma, infections, and inflammation can potentially lead to false-positive troponin results [21]. Initiating inappropriate treatment based solely on elevated troponin levels can lead to severe consequences and missed optimal treatment opportunities. Additionally, the early manifestations in some clinical patients are highly similar to those of ACS, making early differentiation challenging. Given the limitations of current diagnostic tools, the search for novel and more specific biomarkers is crucial.

In recent years, cardiac exosomes have become a highly focused area of research. Under various physiological and pathological conditions, exosomes can selectively load molecular cargo, mediate intercellular communication, and play a significant role in regulating the progression of cardiovascular diseases [29]. Blood samples are easily obtainable in clinical settings, cause minimal harm, and have relatively stable plasma components, making them commonly used in clinical research. Exosomes are abundant in various bodily fluids, including the blood, exhibit tissue specificity, and their membranous structure protects their contents from external environmental interference, making them an excellent reservoir for disease biomarkers [9]. Existing research has demonstrated that under different pathological conditions, the protein composition within exosomes undergoes specific alterations, and the quantity of proteins released into circulation increases [30]. Although the proteome of plasma-derived exosomes is similar to that of the plasma proteome, there are differences in protein content and composition [31]. Therefore, analyzing plasma exosomes has the potential to yield additional and potentially more specific diagnostic information than analyzing plasma alone.

In this study, proteomic technology was applied to identify and quantify plasma exosomal proteins in patients with chest pain, and the results suggested that 24 proteins were significantly different between the two groups, including 11 upregulated and 13 downregulated proteins. GO and KEGG pathway analysis revealed that the differential proteins were significantly enriched in the process of acute inflammatory response and involved in the complement and coagulation cascade pathways. ITIH4, VTN, and HRG were the most significantly upregulated proteins in the discovery cohort, and these proteins were enriched in pathways related to AMI pathophysiology. WB validation of differentially expressed proteins was performed using transferrin as an internal reference gene. Transferrin is the main iron-containing protein in vertebrate plasma, and its expression in serum is relatively stable, making it suitable as an internal reference for serum and plasma samples. Plasma exosome-related studies have used transferrin as an internal reference gene [32]. We performed WB analysis on the DEPs, and the results showed that ITIH4 was significantly elevated in patients with AMI, which may be a potential biomarker for AMI.

ITIH4 is a plasma glycoprotein with a molecular weight of approximately 120 kDa that belongs to the ITI family and is primarily secreted by the liver. The ITI family includes a series of plasma protease inhibitors [33]. ITIH4 expression is significantly related to tumor invasion, metastasis, tissue differentiation, and clinical staging and can be used as a potential diagnostic marker for tumors [34]. It is noteworthy that Huo et al. observed the significant downregulation of total serum ITIH4 levels in patients with coronary heart disease, which was negatively correlated with TNF-α, IL-6, IL-8, IL-17A, C-reactive protein, Gensini score, and serum creatinine. Their study indicated that lower ITIH4 expression levels were associated with more severe coronary stenosis and a higher risk of major adverse cardiovascular events, suggesting that ITIH4 may possess anti-inflammatory properties and play a protective role in the progression of chronic coronary heart disease [35]. A recent study found that total plasma or serum ITIH4 levels are decreased in patients with coronary artery disease and stable angina which associated with coronary thrombus formation, coagulation dysfunction, and an increased risk of adverse cardiovascular events [36]. Additionally, it has been reported that the concentration of ITIH4 in the serum of male patients is significantly upregulated during the acute phase of various pathological states such as acute myocardial infarction, unstable angina, and surgical stimulation [37]. These contradictory expression patterns may reflect the different roles of ITIH4 in different disease stages, sample (total plasma or exosomes), and pathophysiological changes. A study found that ITIH4 is upregulated in bacterial bloodstream infections, suggesting that ITIH4 may be a key inflammatory marker [38]. This study reports firstly that plasma exosomal ITIH4 upregulated significantly in AMI patients, indicating its high diagnostic value. This result is consistent with the role of ITIH4 as the acute-phase marker in previous study [39]. Although studies in stroke have shown that serum ITIH4 is negatively correlated with pro-inflammatory factors such as IL-6, TNF-α, and IL-17A, suggesting the potential anti-inflammatory regulatory function, the upregulation of ITIH4 in our study may be associated with the acute-phase response following myocardial injury. The results show that the expression and function of ITIH4 may exhibit disease specificity across different inflammatory conditions [40]. Given that inflammatory activation critically regulates atherosclerosis progression and correlates with adverse cardiovascular events in patients with coronary artery disease, we hypothesized that post-AMI inflammation drives exosomal ITIH4 release into circulation, resulting in elevated levels of the acute-phase reactive protein ITIH4. The ROC curve results confirmed that the level of plasma exosomal ITIH4 has important reference value for the diagnosis of AMI. Further analysis suggests the significant positive correlation between ITIH4 and cTnI, and their combined application may provide more comprehensive reference for the diagnosis of AMI patients.

Gordon found that VTN knockout mice exhibited enhanced tissue healing and reduced fibrosis after myocardial infarction [41]. A clinical study reported that serum VTN expression levels were elevated in patients with coronary artery disease and correlated with disease severity, identifying VTN as a biomarker for poor cardiovascular prognosis after coronary intervention in ACS patients [42]. These results suggest that VTN may be involved in post‑infarction myocardial fibrosis and cardiac remodeling. Histidine‑rich glycoprotein (HRG) interacts with fibrinogen to modulate coagulation, inflammatory responses, and innate immunity [43]. HRG may act as a negative acute‑phase marker and play an important role in suppressing hemolysis in sepsis [44]. We examined HRG and VTN levels in plasma exosomes and found no significant differences in their expression between the myocardial infarction group and the control group. This may be attributed to the limited sample size, heterogeneous sample sources, and varying stages of the disease.

Our study identifies plasma exosomal ITIH4 as a potential novel biomarker for AMI, with its upregulated expression consistent with an acute-phase response to myocardial injury. If validated in larger cohorts, plasma exosomal ITIH4 has potential as a complementary biomarker to cardiac troponin in AMI, especially when cTn elevation occurs in diagnostically ambiguous contexts such as chronic kidney disease or heart failure. However, we acknowledge that the current study, as a single-center exploratory investigation with a limited sample size, may be constrained in statistics and susceptible to random findings. Additionally, the lack of standardized protocols for exosome isolation and identification currently limits its clinical applicability. Future multicenter, prospective studies with larger cohorts are needed to confirm its diagnostic value with the development of rapid and standardized assays for plasma exosomal protein identification to advance its translation into clinical practice.

## 5. Conclusion

In conclusion, our study identified plasma exosome-derived ITIH4 as a promising diagnostic biomarker of AMI. The association of DEPs with pathways involving the inflammatory response, complement activation, and coagulation cascades provides a biological context consistent with ITIH4’s role as an acute-phase reactant and the underlying pathophysiology of AMI.

## Supporting information

S1 Text Detailed methodology for label-free quantitative proteomics analysis. (DOCX)

S1_raw_images The original uncropped western blot images for Fig 3D and Fig 7A. (PDF)

S1 Fig Characterization of plasma exosomes by NTA. (TIF)

## Abbreviations

ACS: Acute coronary syndrome
AMI: Acute myocardial infarction
AUC: Area under the curve
DEP: Differentially expressed protein
ECG: Electrocardiography
GO: Gene Ontology
ITIH4: Inter-alpha-trypsin inhibitor heavy chain H4
KEGG: Kyoto Encyclopedia of Genes and Genomes
LC-MS/MS: Liquid chromatography-tandem mass spectrometry
MINOCA: Myocardial infarction with non-obstructive coronary arteries
NTA: Nanoparticle tracking analysis
PBS: Phosphate-buffered saline
ROC: Receiver operating characteristic
STEMI: ST-segment elevation myocardial infarction
TEM: Transmission electron microscopy
UA: Unstable angina
WB: Western blot
