# Supplementary material for: Proteomics-based biomarkers of plasma exosomes from patients with acute myocardial infarction
Source: PLoS One. 2026 Mar 10;21(3):e0343804. doi: 10.1371/journal.pone.0343804 (PMC12974808; doi:10.1371/journal.pone.0343804)
Supplement: S1 Text — (DOCX) [file pone.0343804.s001.docx]

1. **Label-free quantitative proteomics**
2. **Protein extraction and digestion**

SDT(4%SDS，100mM Tris-HCl，1mM DTT，pH7.6) buffer was used for sample lysis and protein extraction. The amount of protein was quantified with the BCA Protein Assay Kit (Bio-Rad, USA). Protein digestion by trypsin was performed according to filter-aided sample preparation (FASP) procedure described by Matthias Mann. The digest peptides of each sample were desalted on C18 Cartridges (Empore™ SPE Cartridges C18 (standard density), bed I.D. 7 mm, volume 3 ml, Sigma), concentrated by vacuum centrifugation and reconstituted in 40 µl of 0.1% (v/v) formic acid.

Filter-aided sample preparation (FASP Digestion) procedure：200 μg of proteins for each sample were incorporated into 30 μl SDT buffer (4% SDS, 100 mM DTT, 150 mM Tris-HCl pH 8.0). The detergent, DTT and other low-molecular-weight components were removed using UA buffer (8 M Urea, 150 mM Tris-HCl pH 8.0) by repeated ultrafiltration (Microcon units, 10 kD). Then 100 μl iodoacetamide (100 mM IAA in UA buffer) was added to block reduced cysteine residues and the samples were incubated for 30 min in darkness. The filters were washed with 100 μl UA buffer three times and then 100 μl 25mM NH4HCO3 buffer twice. Finally, the protein suspensions were digested with 4 μg trypsin (Promega) in 40 μl 25mM NH4HCO3 buffer overnight at 37 °C, and the resulting peptides were collected as a filtrate. The peptides of each sample were desalted on C18 Cartridges (Empore™ SPE Cartridges C18 (standard density), bed I.D. 7 mm, volume 3 ml, Sigma), concentrated by vacuum centrifugation and reconstituted in 40 µl of 0.1% (v/v) formic acid. The peptide content was estimated by UV light spectral density at 280 nm using an extinctions coefficient of 1.1 of 0.1% (g/l) solution that was calculated on the basis of the frequency of tryptophan and tyrosine in vertebrate proteins.

1. **SDS-PAGE**

20 µg of protein for each sample were mixed with 5X loading buffer respectively and boiled for 5 min. The proteins were separated on 12.5% SDS-PAGE gel (constant current 14 mA, 90 min). Protein bands were visualized by Coomassie Blue R-250 staining.

1. **LC-MS/MS analysis**

LC-MS/MS analysis was performed on a Q Exactive mass spectrometer (Thermo Scientific) that was coupled to Easy nLC (Proxeon Biosystems, now Thermo Fisher Scientific) for 60/120/240 min. The peptides were loaded onto a reverse phase trap column (Thermo Scientific Acclaim PepMap100, 100 μm*2 cm, nanoViper C18) connected to the C18-reversed phase analytical column (Thermo Scientific Easy Column, 10 cm long, 75 μm inner diameter, 3μm resin) in buffer A (0.1% Formic acid) and separated with a linear gradient of buffer B (84% acetonitrile and 0.1% Formic acid) at a flow rate of 300 nl/min controlled by IntelliFlow technology. The mass spectrometer was operated in positive ion mode. MS data was acquired using a data-dependent top10 method dynamically choosing the most abundant precursor ions from the survey scan (300–1800 m/z) for HCD fragmentation. Automatic gain control (AGC) target was set to 3e6, and maximum inject time to 10 ms. Dynamic exclusion duration was 40.0 s. Survey scans were acquired at a resolution of 70,000 at m/z 200 and resolution for HCD spectra was set to 17,500 at m/z 200, and isolation width was 2 m/z. Normalized collision energy was 30 eV and the underfill ratio, which specifies the minimum percentage of the target value likely to be reached at maximum fill time, was defined as 0.1%. The instrument was run with peptide recognition mode enabled.

1. **Identification and quantitation of proteins**

The MS raw data for each sample were combined and searched using the MaxQuant 1.5.3.17 software for identification and quantitation analysis. Related parameters and instructions are as follows:

Table Maxquant identification and quantitation indexes

| Item | Value |
| --- | --- |
| Enzyme | Trypsin |
| Max Missed Cleavages | 2 |
| Fixed modifications | Carbamidomethyl (C) |
| Variable modifications | Oxidation (M) |
| Main search | 6 ppm |
| First search | 20ppm |
| MS/MS Tolerance | 20ppm |
| Database | https://www.uniprot.org/proteomes/UP000005640 |
| Database pattern | Reverse |
| Include contaminants | True |
| protein FDR | ≤0.01 |
| Peptide FDR | ≤0.01 |
| Peptides used for protein quantification | Use razor and unique peptides |
| Time window (match between runs) | 2min |
| protein quantification | LFQ |
| min. ratio count | 1 |
