# Supplementary material for: Proteomics-based biomarkers of plasma exosomes from patients with acute myocardial infarction
Source: PLoS One. 2026 Mar 10;21(3):e0343804. doi: 10.1371/journal.pone.0343804 (PMC12974808; doi:10.1371/journal.pone.0343804)
Supplement: S1_raw_images — (PDF) [file pone.0343804.s002.pdf]

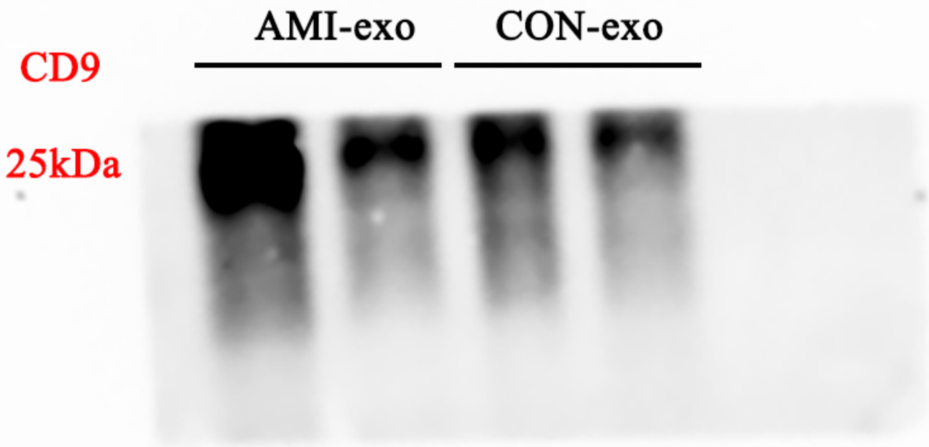

The original, uncropped western blot image of CD9 for Figure 3D.

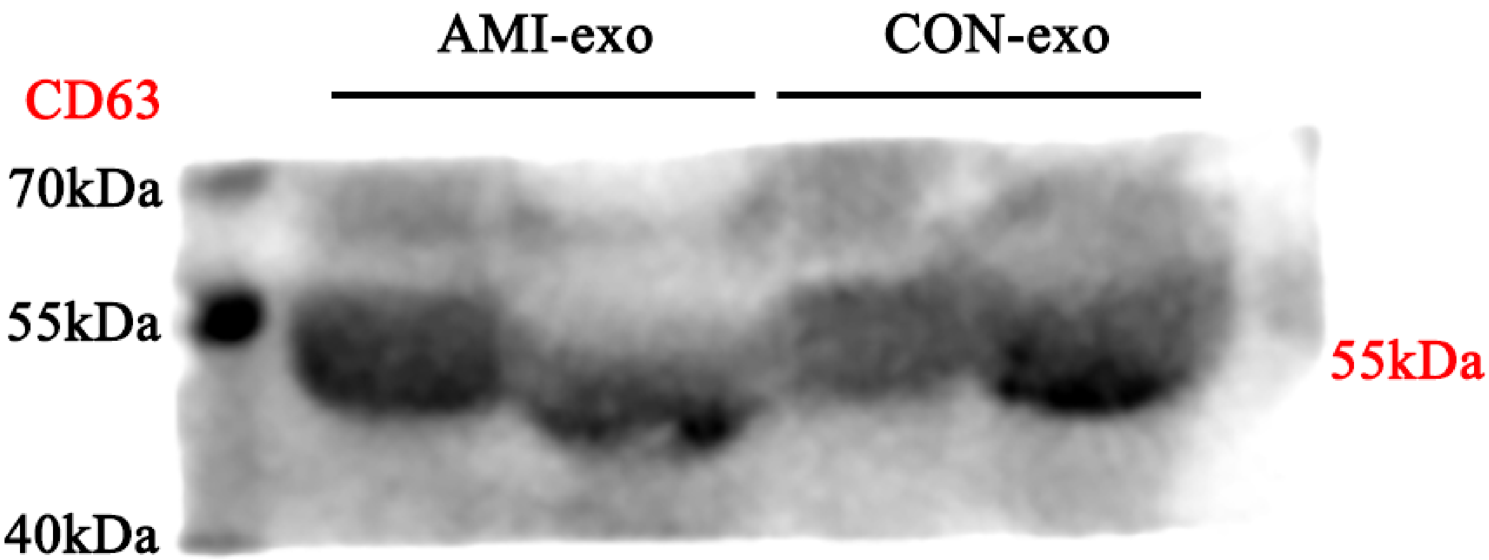

The original, uncropped western blot image of CD63 for Figure 3D.

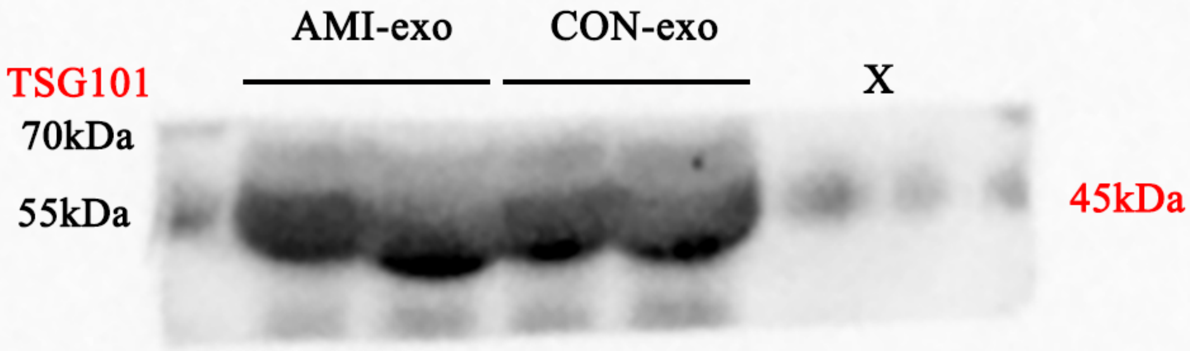

The original, uncropped western blot image of TSG101 for Figure 3D.

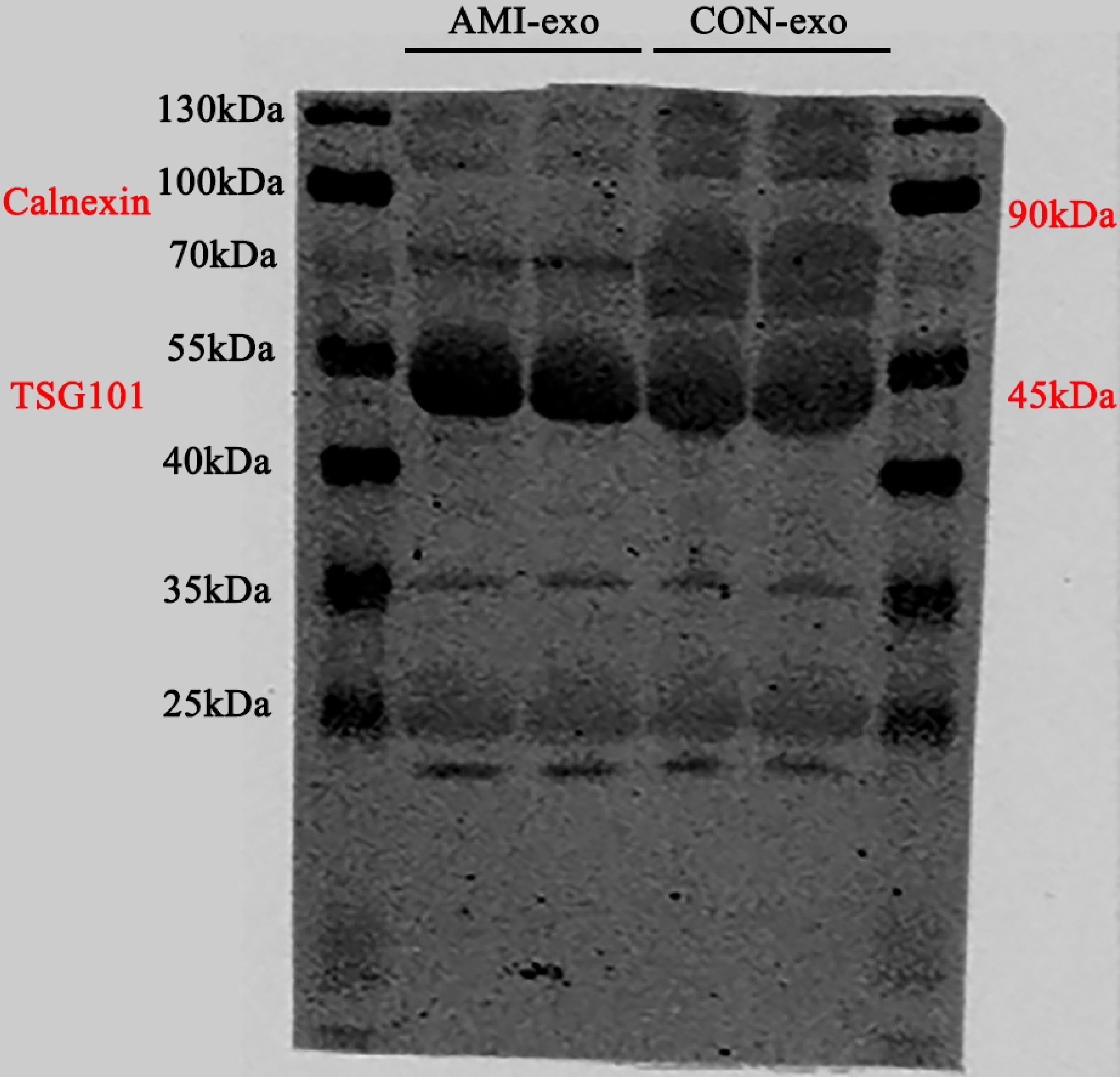

The original, uncropped western blot image of TSG101 and Calnexin for Figure 3D.

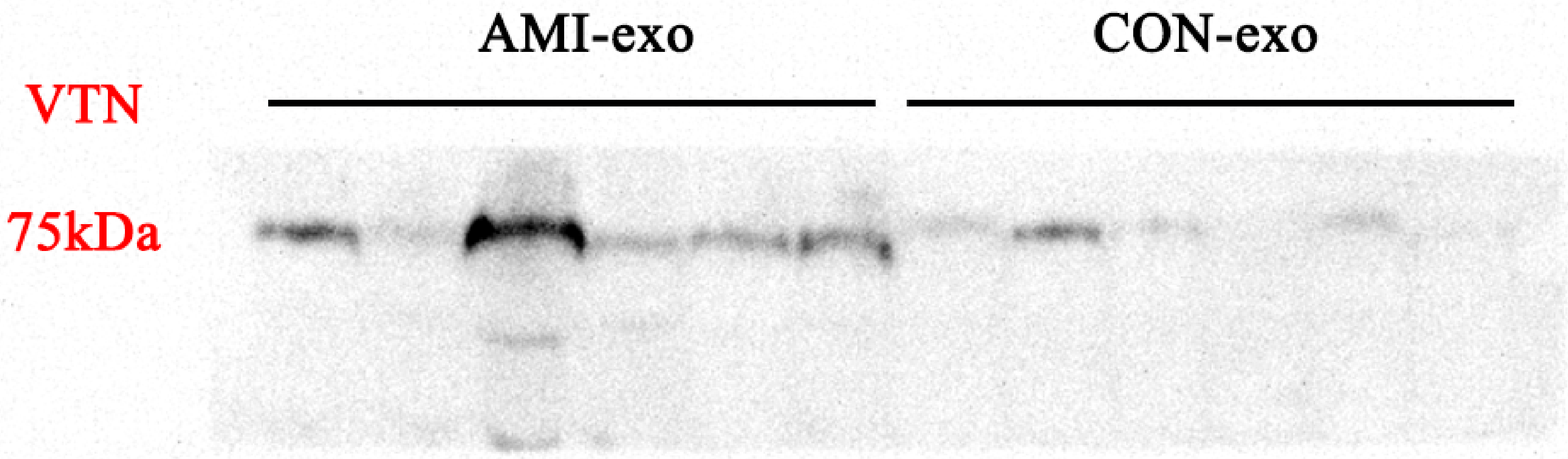

The original, uncropped western blot image of VTN for Figure 7A.

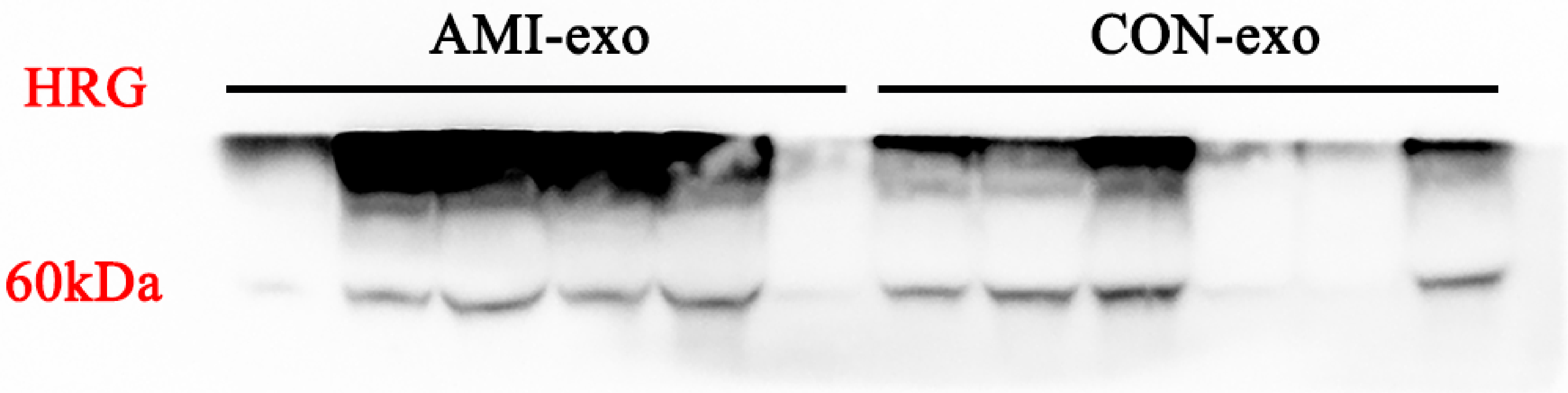

The original, uncropped western blot image of HRG for Figure 7A.

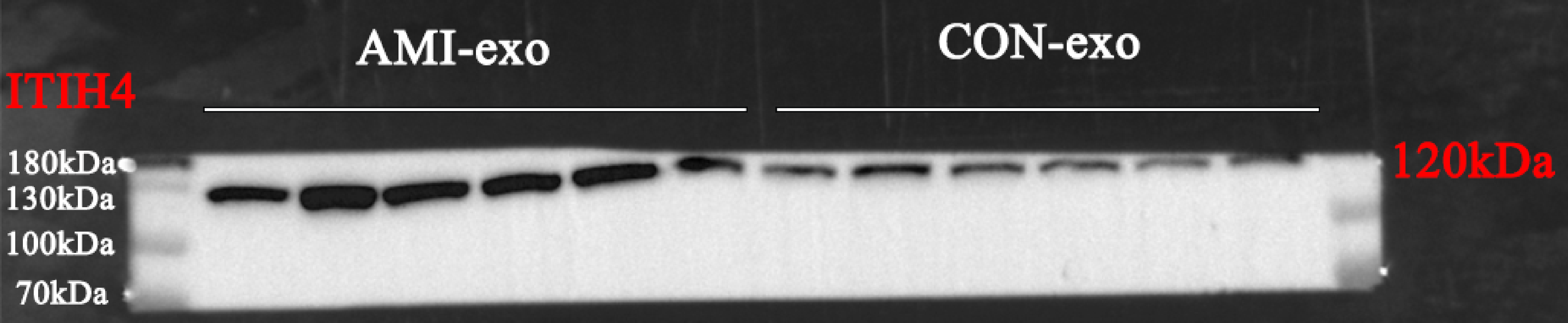

The original, uncropped western blot image of ITIH4 for Figure 7A.

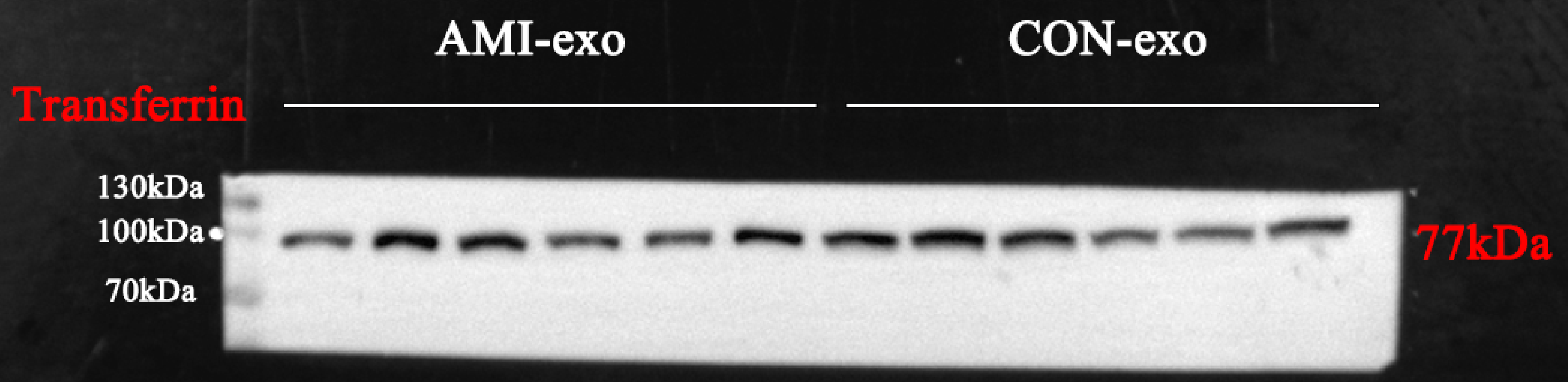

The original, uncropped western blot image of Transferrin for Figure 7A.
