# Supplementary figures and images for: Proteomics-based biomarkers of plasma exosomes from patients with acute myocardial infarction
Source: PLoS One. 2026 Mar 10;21(3):e0343804. doi: 10.1371/journal.pone.0343804 (PMC12974808; doi:10.1371/journal.pone.0343804)

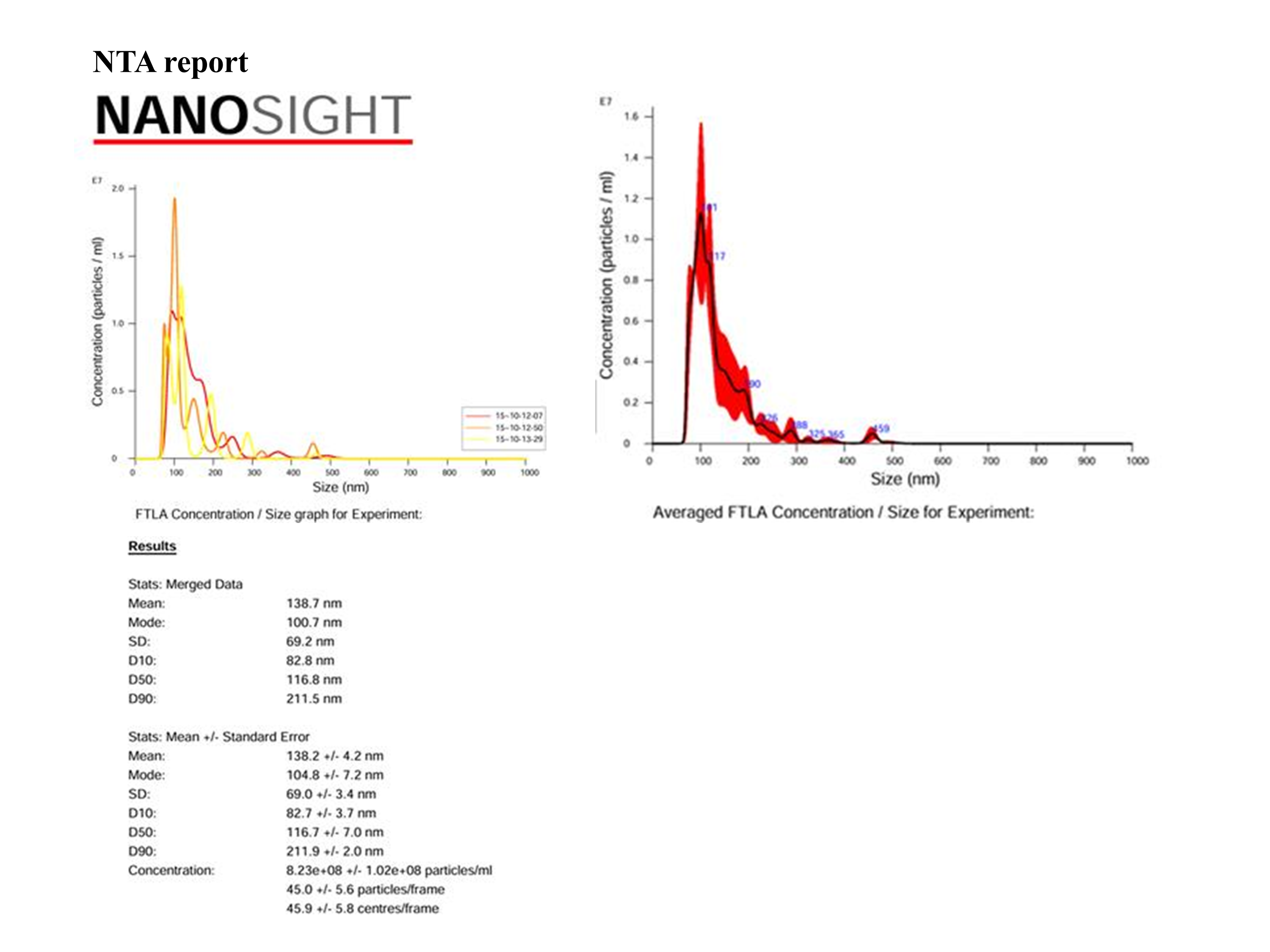

Supplement: S1 Fig — (TIF) [file pone.0343804.s003.tif]
